# Resistance to doxorubicin therapy in breast cancer cells could be attenuated by ligustilide: impact on autophagy and LncRNA H19

**DOI:** 10.1186/s12906-026-05397-0

**Published:** 2026-05-13

**Authors:** Eman A. Amr, Ola A. El-Feky, Eman G. Khedr, Nahla E. El-Ashmawy

**Affiliations:** 1 Department of Biochemistry, Faculty of Pharmacy, Tanta University, Tanta, El-Gharbia 31527 Egypt; 2 Department of Pharmacology and Biochemistry, Faculty of Pharmacy, The British University in Egypt, El Sherouk City, Cairo, 11837 Egypt

**Keywords:** Autophagy, BC, Doxorubicin, Ligustilide, ERα, LncRNA H19

## Abstract

**Background:**

Resistance to DOX in breast cancer (BC) treatment is attributed to multiple mechanisms. The potential of autophagy inhibition in mitigating DOX resistance in BC has yet to be clarified. Despite showing anti-tumorigenic activity, the role of ligustilide (LIG) in BC treatment remains limited.

**Aim:**

To evaluate LIG-mediated autophagy inhibition in DOX-treated MCF-7, MCF-7/DOX, and MDA-MB-231 cells, and investigate LncRNA H19’s role in BC resistance.

**Methods:**

The three cell lines were treated with DOX, LIG, or both of them. Autophagic flux and expression of ERα, metastasis-associated protein (MTA1) complex components, autophagy-related 7 gene (*ATG7*), multidrug resistance gene 1 (*MDR1*) and LncRNA H19 were assessed.

**Key findings:**

Both individual treatments showed dose-dependent cytotoxicity. When combined together, LIG increased DOX cytotoxicity in MCF-7/DOX and MDA-MB-231. LIG inhibited DOX-induced autophagic flux, indicated by elevated LC3BII and p62 levels, and reversed *MDR1* in MCF-7/DOX and MDA-MB-231. LIG monotherapy upregulated ERα in MCF-7/DOX and restored its expression in MDA-MB-231. In MCF-7/DOX, combined treatment upregulated ERα and downregulated LncRNA H19 relative to DOX monotherapy, whereas in MDA-MB-231, it upregulated LncRNA H19 compared with DOX monotherapy.

**Conclusion:**

LIG mitigated DOX-induced resistance through inhibiting autophagy, restoring ERα expression, and downregulating LncRNA H19 in the DOX-resistant BC cell line, thereby presenting a potential therapeutic approach in BC treatment.

## Introduction

Breast cancer (BC) ranks as the most prevalent cancer type and the top contributor to cancer-related deaths in women around the world [1]. Treatment for this type of cancer is difficult since it has several subtypes that vary in genetics, pathology, and clinical presentation. Approximately 70% of BC patients have the estrogen receptor-positive (ER + ve) subtype, which responds better to chemotherapy or antihormonal therapy than the ER-negative (ER-ve) subtype [2]. About 15–20% of BCs are the triple-negative BC (TNBC) subtype, i.e., they are estrogen, progesterone, and human epidermal growth factor receptor 2 negative (ER-, PR-, and HER2-ve). This subtype is characterized by highly aggressive clinical behavior relative to BCs expressing ER, PR, and HER2 [3].

Chemoresistance is the primary driver of BC recurrence, relapse, and mortality, and despite advancements in treatment, it continues to hinder the effective management of all BC subtypes [4]. Generally, there are two categories of chemoresistance: intrinsic resistance (preexisting) and acquired resistance (induced by the drugs) [5]. Chemoresistance can be triggered by a wide range of identified processes, including metabolic drug deactivation, structural changes in drug targets, enhanced drug efflux, genetic mutation, epigenetic modifications, deregulation of apoptosis, DNA repair, and activation of survival mechanisms, including autophagy [5, 6].

Autophagy is a homeostatic recycling mechanism that helps all cells maintain cellular turnover, eliminate damaged organelles, and adapt to physiological and pathological conditions. Autophagy has a paradoxical role in cancer; in early stages of carcinogenesis, it may have a tumor-suppressive effect by breaking down potentially dangerous agents or damaged organelles. However, in late stages of cancer, autophagy plays a tumor-promotive role by sustaining tumor survival in stressful microenvironments. Besides, autophagy contributes notably to resistance to several types of cancer therapy, representing a significant challenge to successful treatment. In BC, autophagy is induced by different therapies and plays a variety of roles, either suppressing or increasing treatment efficacy depending on the cell type [3, 6].

Doxorubicin (DOX) is a naturally occurring anthracycline antibiotic typically used as a neoadjuvant therapy in the treatment of ER + ve BC and TNBC subtypes [7]. It triggers the production of free radicals leading to DNA damage, disrupts topoisomerase II activity, and intercalates with the DNA duplex in rapidly dividing cells, which subsequently prevents DNA replication and mRNA transcription processes [8]. Long-term DOX administration frequently results in life-threatening cardiotoxicity and DOX resistance, which limit the effectiveness of BC treatment. The efficient treatment of BC would significantly improve if DOX resistance were eliminated and its harmful cardiac effects were reduced [5].

Metastasis-associated protein 1 (MTA1) is an integral part of the nucleosome remodeling and histone deacetylase (NuRD) complex that represses the transactivation activity of ERα [9]. MTA1 is overexpressed in BC and has a strong correlation with metastasis and invasion [10, 11]. A comprehensive proteomic study of the MTA1 complex showed that the complex includes the transcription factor AP-2 g (TFAP2C) and the IFN**-γ-**inducible protein 16 (IFI16). The complexes formed by MTA1 with either TFAP2C or IFI16 in BC might be involved in modulating estrogen receptor alpha (*ESR1*) gene expression epigenetically, thereby influencing responsiveness to anti-estrogen therapy [12].

Long noncoding RNA (LncRNA) H19 is an essential regulator of development with emerging roles in cancer progression. It is the first identified oncogenic LncRNA implicated at several stages of carcinogenesis. In BC, LncRNA H19 was defined as an estrogen receptor modulator associated with both cancer development and chemoresistance [13, 14].

Ligustilide (LIG), a phthalide compound derived from *Radix Angelica sinensis*, is commonly utilized in traditional Chinese medicine for the management of BC and promotion of cardiovascular health [9]. When combined with DOX treatment, this novel adjuvant therapy showed a typical cardioprotective action against DOX-induced oxidative stress [15]. LIG has shown anti-tumor activity across various human cancers, including BC, in addition to its anti-inflammatory and antioxidant properties [16]. LIG was found to sensitize resistant ER + ve BC through inhibiting autophagy, while restoring anti-estrogen sensitivity of ER-ve BC by reversing epigenetic repression of ERα [9, 17].

We hypothesized that co-treatment of BC cells with the cytotoxic doxorubicin and LIG would result in a greater therapeutic outcome than either monotherapy. For our knowledge, this is the first study that aimed to elucidate the impact of LIG and/or DOX on autophagic response in ER-ve (MDA-MB-231), sensitive ER + ve (MCF-7), and resistant ER + ve (MCF-7/DOX) cell lines. Additionally, the role of LncRNA H19 in BC resistance, and whether it could be a target of therapy, was also evaluated.

## Materials and methods

We followed all the guidelines approved by the Research Ethical Committee at the Faculty of Pharmacy, Tanta University in Egypt (TP/RE/4/25 p-002).

Clinical trial number: not applicable.

### Cell lines and culture conditions

The human breast adenocarcinoma cell line (MCF-7), the human triple-negative BC cell line (MDA-MB-231), and the human doxorubicin resistant BC cell line (MCF-7/DOX) were obtained from Nawah Scientific Inc. (Mokatam, Cairo, Egypt). Cells (1 × 10⁶ per flask) were seeded in 25 cm² tissue culture flasks, maintained in Dulbecco’s modified Eagle medium (DMEM) with 1% of penicillin/streptomycin/amphotericin B (Gibco, USA) and 10% of fetal bovine serum (FBS) (Sigma-Aldrich, USA), then incubated for 24 h under standard conditions (37 °C, 5% CO₂, humidified atmosphere) to allow exponential growth prior to treatment [3].

### Drugs

Doxorubicin hydrochloride powder with a purity of 98.0–102.0%, ligustilide liquid with a purity of ≥ 96%, and dimethylsulfoxide (DMSO) were obtained from Sigma-Aldrich (USA). DOX stock solution (100 µM) was prepared in culture medium, then serially diluted with complete medium to achieve the required working concentrations [18, 19]. LIG was dissolved in DMSO to prepare a (0.5 M) stock solution, then kept at − 20˚C. LIG was freshly diluted in culture medium from its stock solution immediately prior to use to obtain the needed working concentrations. All preparations contained a final DMSO concentration of ≤ 0.1%, a nontoxic level for cells [20, 21].

### Cytotoxicity assay

The Sulforhodamine B (SRB) assay was performed to assess cell viability [22]. Briefly, a 100 µL aliquot of cell suspension containing 5 × 10³ cells was added to each well of a 96-well plate and incubated in complete medium for 24 h. Another 100µL aliquot of medium with different drug concentrations was added to the wells. Following 72 h of treatment, the culture medium was removed, and 150µL of 10% trichloroacetic acid (TCA) (Sigma-Aldrich, USA) was added to fix the cells and incubated at 4 °C for 1 h. The TCA was aspirated, followed by five washes with distilled water to remove any residual fixative. 70µL aliquots of 0.4% (w/v) SRB solution (Sigma-Aldrich, USA) were added to the wells, followed by incubation in the dark at room temperature for 10 min. Plates were rinsed three times with 1% acetic acid (Thermo Fisher Scientific, USA) and then left to dry in air during the night. Then, 150µLs of 10mM tris(hydroxymethyl)aminomethane (Thermo Fisher Scientific, USA) was added to dissolve the protein-bound SRB dye, and absorbance was measured at 540 nm with a FLUOstar Omega microplate reader (BMG LABTECH^®^, Ortenberg, Germany). Each drug concentration was tested in triplicate. The following equation was used to determine cell viability: (Abs s / Abs c) × 100, where Abs s refers to the absorbance of cells incubated with the drug, while Abs c corresponds to that of untreated cells.

Initially, SRB was performed for DOX and LIG for 72 h in all tested cell lines, each tested alone with different concentration ranges of (0.01, 0.1, 1, 10, and 100 µM) for DOX and (0.05, 0.5, 5, 50, and 500 µM) for LIG for assessing cytotoxic effects and determining the half maximal inhibitory concentration (IC50). Based on the results obtained, DOX concentration with no cytotoxic effect on MCF-7/DOX was chosen to estimate the potential therapeutic benefit of its co-treatment with LIG. Accordingly, 0.01 µM of DOX was selected to be used in combination with 50 µM of LIG. Then, the three tested cells were exposed to 0.01 µM of DOX, 50 µM of LIG, or both of them for 72 h followed by performing the SRB assay.

### Evaluation of drug resistance

The fold resistance to DOX and LIG in MCF-7/DOX and MDA-MB-231 cell lines was evaluated by calculating the ratio of each drug’s IC50 in the resistant cell line to its IC50 in the sensitive MCF-7 cell line [6].

### Assessment of the autophagy markers

Human microtubule-associated protein 1 light chain-3B II (LC3BII) and the ubiquitin binding protein p62 (known as sequestosome 1; SQSTM1) are widely recognized markers used to evaluate autophagic flux activity [3]. Human LC3BII and p62 protein concentrations were measured by enzyme-linked immunosorbent assay (ELISA) kits (SunLong Biotech, China) according to the provided protocol, and the concentrations were reported in ng/L.

### Assessment of estrogen receptor alpha protein and metastasis-associated protein 1 complex components

The levels of ERα protein and the MTA1 complex components (MTA1, IFI16, and TFAP2C) were assessed by ELISA kits (SunLong Biotech, China) following the provided protocol, and the results were reported in ng/L.

### Quantitative reverse-transcription polymerase chain reaction (qRT-PCR)

The qRT-PCR was used to determine the gene expression of LncRNA H19, multidrug resistance 1 (*MDR1*), autophagy-related 7 (*ATG7*), *ESR1*, *MTA1*, *IFI16*, and *TFAP2C* genes in harvested cells. The RNeasy^®^ Mini Kit (QIAGEN, Germany) was utilized for total RNA extraction from the collected cells’ lysate in accordance with the provided protocol. The QuantiTect^®^ reverse transcription (QIAGEN, Germany) was utilized for complementary DNA (cDNA) synthesis from the extracted RNA. Using the QuantiTect^®^ SYBR Green PCR Kit (QIAGEN, Germany), generated cDNA was utilized for the amplification and quantification of the genes. Target gene threshold cycle (Ct) values were compared to the Ct value of the housekeeping gene, glyceraldehyde-3-phosphate dehydrogenase (GAPDH), and presented as fold change. Using Rotor-Gene Q 5plex (QIAGEN, Germany), the real-time PCR program was initiated with heat activation at 95 °C for 15 min, then run through 45 cycles at 94 °C for 15 s, 55 °C for 30 s, and 72 °C for 30 s. Gene expression fold change was determined according to the 2^−ΔΔCt^ method [3]. The primer sequences applied in this experiment are listed in Table 1.

**Table 1:** Primer sequences used for qRT-PCR

Gene	Primer sequences	
	Forward sequence (5’-3’)	Reverse sequence (5’-3’)
*MDR1*	GCTGTCAAGGAAGCCAATGCCT	TGCAATGGCGATCCTCTGCTTC
*ATG7*	GATCCGGGGATTTCTTTCACG	CAGCAATGTAAGACCAGTCAAGT
*MTA1*	CACGCACATCAGGGGCAA	GTGCGAAGGTGCCCACA
*ESR1*	ACTGTAGCAGAGTATCTGGTGA	GGTCTGCAAGGAATGTTCCTA
*IFI16*	ACAAACCCGAGAAACAATGACC	GCATCTGAGGAGTCCGAAGA
*TFAP2C*	GAAGAGGACTGCGAGGATCG	GCTGATATTCGGCGACTCCA
LncRNA H19	TGGCCATGAAGATGGAGTCG	TACAACCACTGCACTACCTG
*GAPDH*	GTCTCCTCTGACTTCAACAGCG	ACCACCCTGTTGCTGTAGCCAA

### Statistical analysis

Statistical Package for the Social Sciences (SPSS) 28.0 for Windows SPSS Inc. (Chicago, IL, USA) was used. The normality of distribution for the analyzed variables was tested using the Shapiro test. The collected data were presented as mean and standard deviation (SD) for parametric data. The statistical significance among groups was assessed using the one-way analysis of variance (ANOVA) test, and subsequently, the post-hoc Tukey test was performed as appropriate for parametric data. Comparisons between two groups were performed using Student’s t-test. The p-value was considered significant at *p* < 0.05.

## Results

### Effect of DOX and LIG on cell viability

**Fig. 1:**
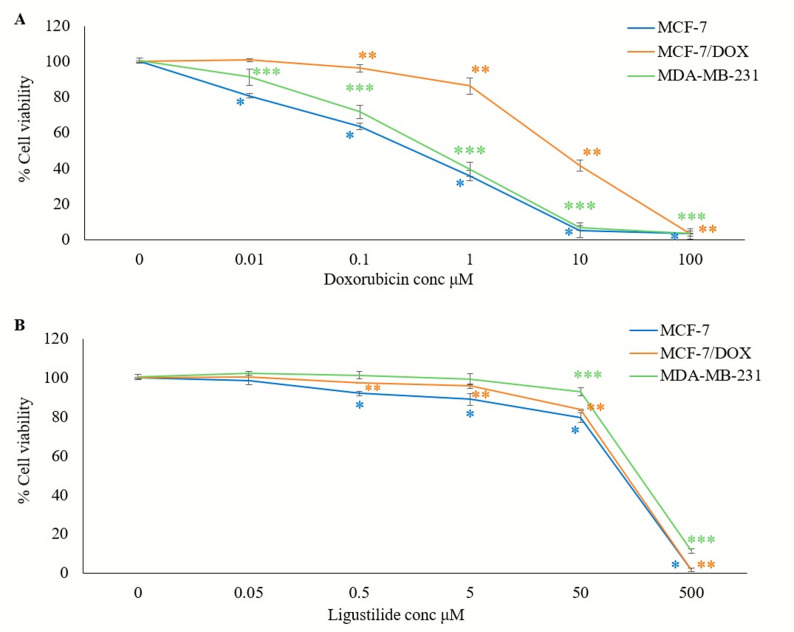
Cell viability of MCF-7, MCF-7/DOX, and MDA-MB-231 cell lines was evaluated using the SRB assay after 72 h of treatment with (0.01–100µM) Doxorubicin (**A**) and (0.05–500µM) Ligustilide (**B**). Values are presented as mean ± SD (*n* = 3 replicates per concentration). *: Statistically significant compared to MCF-7 untreated control; **: Statistically significant compared to MCF-7/DOX untreated control; ***: Statistically significant compared to MDA-MB-231 untreated control

The SRB assay was used to determine the cell viability relative to the untreated controls. DOX monotherapy reduced cell viability in a dose-dependent manner across all tested concentrations in both MCF-7 and MDA-MB-231 cells, with viability ranging from 80.71 ± 1.27% to 3.41 ± 1.54% in MCF-7 cells and from 91.33 ± 4.47% to 3.2 ± 2.9% in MDA-MB-231 cells. A comparable dose-dependent decline was observed in MCF-7/DOX cells over 0.1–100µM (96.39 ± 2.1% to 3.4 ± 0.3%), whereas 0.01µM DOX had no significant effect, indicating resistance at low concentration (Fig. 1A). The IC50 values were 0.25, 0.42, and 6.78µM for MCF-7, MDA-MB-231, and MCF-7/DOX cells, respectively, corresponding to 1.68 fold and 27.12 fold higher resistance in MDA-MB-231 and MCF-7/DOX cells, respectively, relative to MCF-7.

LIG monotherapy also decreased cell viability in a dose-dependent manner. In MCF-7 and MCF-7/DOX cells (0.5–500 µM), viability declined from 92.06 ± 1.0% to 1.68 ± 0.99% and from 97.43 ± 0.3% to 1.70 ± 0.89%, respectively. In the MDA-MB-231 cells (50–500 µM), viability decreased from 93.06 ± 2.03% to 11.5 ± 1.15% (Fig. 1B). The IC50 values were 95.1, 97.88, and 181.48µM for MCF-7, MCF-7/DOX, and MDA-MB-231 cells, respectively, indicating a 1.9 fold increase in resistance in MDA-MB-231 cells and minimal change in MCF-7/DOX cells (1.02 fold) relative to MCF-7.

**Fig. 2:**
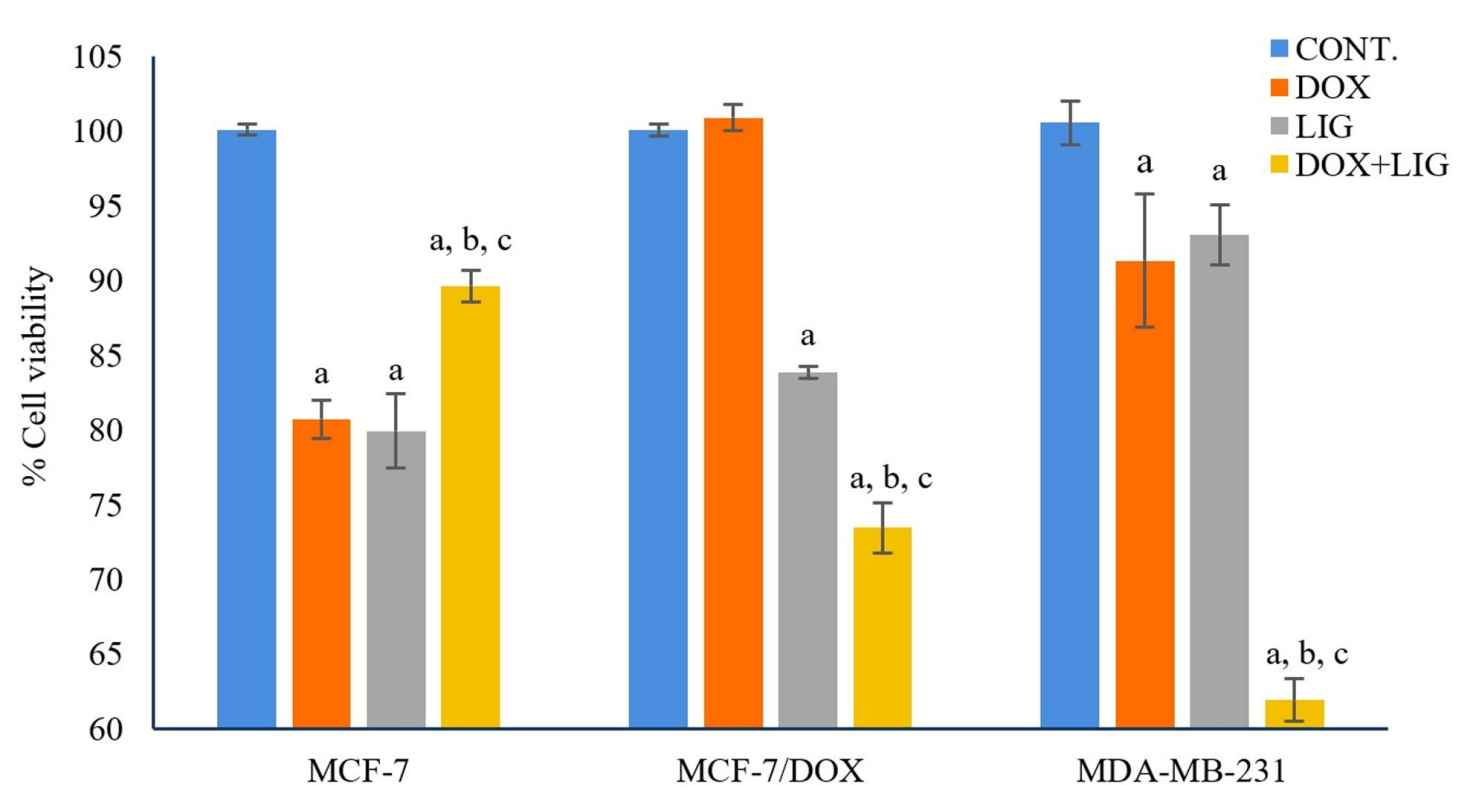
Impact of various treatments on cell viability. Values are presented as mean ± SD (*n* = 3 per group). CONT: Untreated control; DOX: Doxorubicin (0.01 µM); LIG: Ligustilide (50 µM). a: Statistically significant compared to respective untreated control; b: Statistically significant compared to respective DOX-treated cells; c: Statistically significant compared to respective LIG-treated cells

Co-treatment with 0.01 µM DOX and 50 µM LIG produced cell line-dependent effects (Fig. 2). In MCF-7 cells, the combination resulted in 89.65 ± 1.07% viability relative to the untreated control, indicating minimal cytotoxicity compared to DOX (80.71 ± 1.27%) or LIG (79.94 ± 2.49%) monotherapy, suggesting an antagonistic interaction.

In contrast, the combination of LIG with DOX enhanced DOX cytotoxicity in both MCF-7/DOX and MDA-MB-231 cells, reducing cell viability to 73.46 ± 1.67% and 61.93 ± 1.43%, respectively, relative to the untreated controls. Conversely, individual treatments of DOX (0.01µM) or LIG (50µM) produced higher viability: 100.89 ± 0.86% and 83.83 ± 0.39% in MCF-7/DOX, and 91.33 ± 4.47% and 93.06 ± 2.03% in MDA-MB-231, respectively.

Overall, the combined therapy significantly enhanced cytotoxicity in MCF-7/DOX and MDA-MB-231 cells compared with either monotherapy.

### Impact of DOX and LIG on autophagy markers

**Fig. 3:**
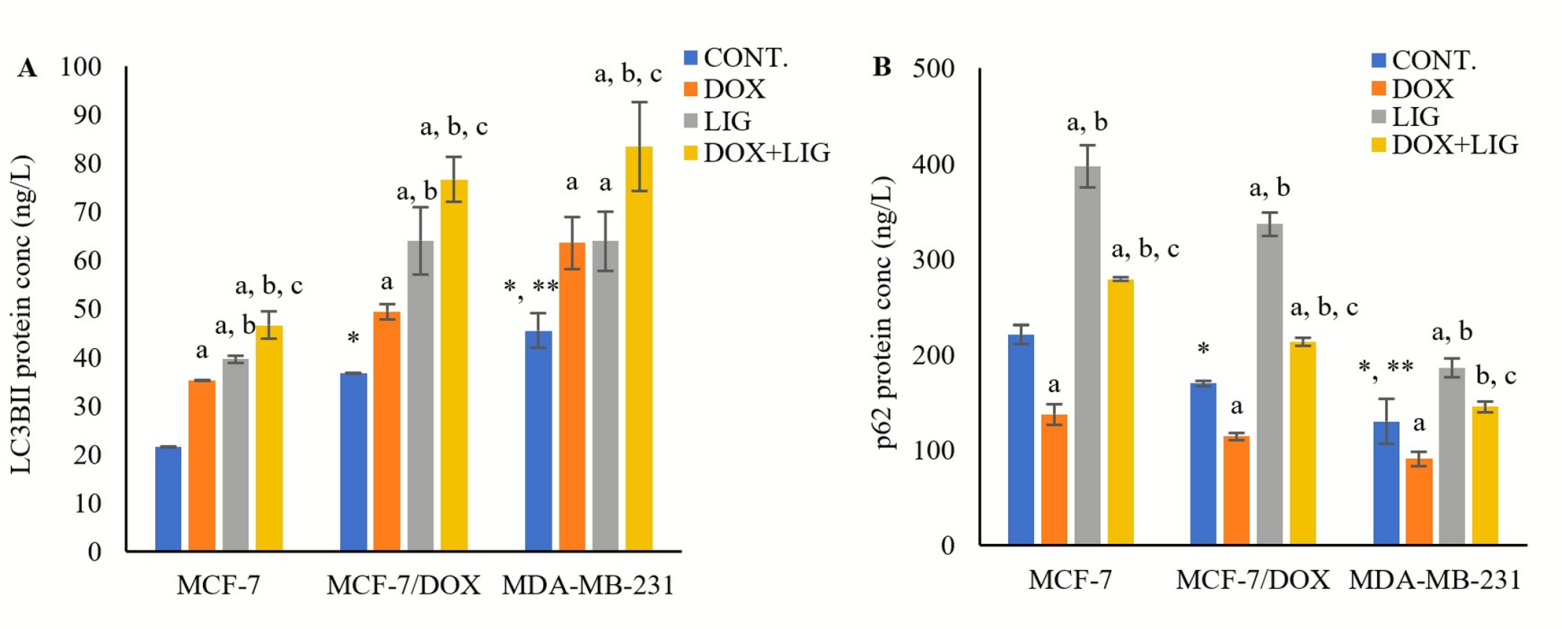
Concentrations of LC3BII (**A**) and p62 (**B**) in MCF-7, MCF-7/DOX, and MDA-MB-231 cell lines evaluated by ELISA after 72 h of treatment with (0.01µM) Doxorubicin, (50µM) Ligustilide, or a combination of both drugs. Values are presented as mean ± SD (*n* = 3 per group). CONT.: Untreated control; DOX: Doxorubicin; LIG: Ligustilide. *: Statistically significant compared to MCF-7 untreated control; **: Statistically significant compared to MCF-7/DOX untreated control. a: Statistically significant compared to respective untreated control; b: Statistically significant compared to respective DOX-treated cells; c: Statistically significant compared to respective LIG-treated cells

The basal levels of the autophagy-related markers LC3BII and p62 proteins were measured. Basal LC3BII protein levels were significantly higher in MCF-7/DOX cells (36.79 ± 0.02 ng/L, *p* < 0.001) relative to MCF-7 cells (21.52 ± 0.14 ng/L). and in MDA-MB-231 cells (45.51 ± 3.48 ng/L) relative to both MCF-7 (*p* < 0.001) and MCF-7/DOX cells (*p* < 0.01) (Fig. 3A).

Both DOX and LIG monotherapies significantly increased LC3BII protein levels compared with untreated controls in all cell lines: MCF-7 (35.32 ± 0.02 ng/L and 39.67 ± 0.77 ng/L, respectively, *p* < 0.001), MCF-7/DOX (49.37 ± 1.58 ng/L, *p* < 0.05 and 64.02 ± 6.92 ng/L, *p* < 0.001, respectively), and MDA-MB-231 (63.56 ± 5.35 ng/L and 63.94 ± 6.15 ng/L, respectively, *p* < 0.05). The DOX + LIG combined therapy further and significantly increased LC3BII protein levels compared with either monotherapy in MCF-7 (46.61 ± 2.77 ng/L, *p* < 0.001 and *p* < 0.01, respectively), MCF-7/DOX (76.63 ± 4.65 ng/L, *p* < 0.01 and *p* < 0.05, respectively), and MDA-MB-231 (83.4 ± 9.14 ng/L, *p* < 0.05) (Fig. 3A).

Basal p62 protein levels were significantly lower in MCF-7/DOX cells (169.94 ± 2.49 ng/L, *p* < 0.05) relative to the MCF-7 cells (221.05 ± 9.76 ng/L), and in MDA-MB-231 cells (130.00 ± 23.57 ng/L) compared to both MCF-7 cells (*p* < 0.001) and MCF-7/DOX cells (*p* < 0.05) (Fig. 3B).

DOX monotherapy significantly lowered p62 protein levels in all cell lines: MCF-7 (137.44 ± 11.00 ng/L, *p* < 0.001), MCF-7/DOX (114.55 ± 3.96 ng/L, *p* < 0.001), and MDA-MB-231 (90.94 ± 7.53 ng/L, *p* < 0.05) compared with untreated controls. On the contrary, LIG monotherapy significantly increased p62 protein levels in all cell lines: MCF-7 (397.00 ± 22.33 ng/L, *p* < 0.001), MCF-7/DOX (336.88 ± 12.13 ng/L, *p* < 0.001), and MDA-MB-231 (186.05 ± 9.94 ng/L, *p* < 0.01) compared to the untreated controls.

The DOX + LIG combined therapy significantly elevated p62 levels compared with DOX monotherapy in MCF-7, MCF-7/DOX, and MDA-MB-231 cells (278.72 ± 1.83 ng/L, 213.83 ± 4.40 ng/L, *p* < 0.001, and 145.61 ± 5.87 ng/L, *p* < 0.01, respectively). In contrast, the combination significantly lowered p62 levels relative to LIG monotherapy in all three cell lines: MCF-7, MCF-7/DOX (*p* < 0.001), and MDA-MB-231 (*p* < 0.05) (Fig. 3B).

### Effect of DOX and LIG on multidrug resistance 1 gene expression

**Fig. 4:**
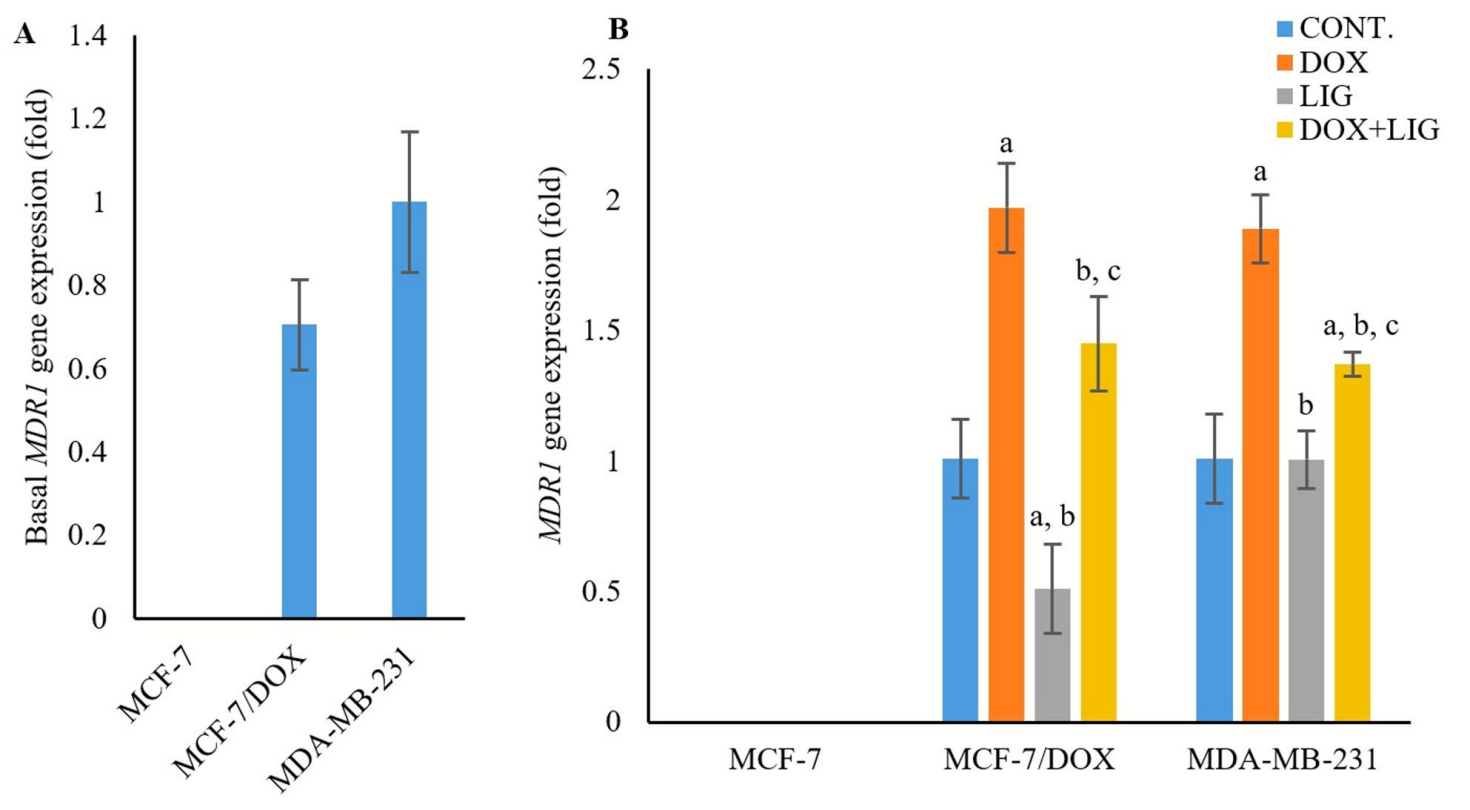
Basal expression of *MDR1* gene (**A**) and expression of *MDR1* gene in the treated cell line groups (**B**) evaluated by PCR after 72 h of treatment with (0.01 µM) Doxorubicin, (50 µM) Ligustilide, or a combination of both drugs. Values are presented as mean ± SD (*n* = 3 per group). CONT: Untreated control; DOX: Doxorubicin; LIG: Ligustilide. a: Statistically significant compared to respective untreated control; b: Statistically significant compared to respective DOX-treated cells; c: Statistically significant compared to respective LIG-treated cells

Basal *MDR1* mRNA expression in MCF-7/DOX cells (0.706 ± 0.108 fold, *p* > 0.05) did not differ significantly compared with MDA-MB-231 cells (Fig. 4A).

DOX monotherapy significantly upregulated *MDR1* expression in MCF-7/DOX and MDA-MB-231 (1.97 ± 0.17 fold and 1.89 ± 0.13 fold, respectively, *p* < 0.001) compared with untreated controls. In contrast, LIG monotherapy significantly downregulated *MDR1* expression in MCF-7/DOX (0.51 ± 0.17 fold, *p* < 0.05), but had no significant effect in MDA-MB-231 (1.0 ± 0.11 fold, *p* > 0.05), compared to respective untreated controls. The DOX + LIG combined therapy significantly downregulated *MDR1* expression relative to DOX monotherapy in both MCF-7/DOX and MDA-MB-231 cells (1.45 ± 0.18 fold, *p* < 0.05, and 1.37 ± 0.04 fold, *p* < 0.01, respectively), yet *MDR1* expression remained higher than those observed with LIG monotherapy (*p* < 0.001 and *p* < 0.05, respectively). Notably, *MDR1* gene expression was undetectable in MCF-7 cells, regardless of treatment or control conditions (Fig. 4B).

### Impact of DOX and LIG on autophagy-related 7 gene expression

**Fig. 5:**
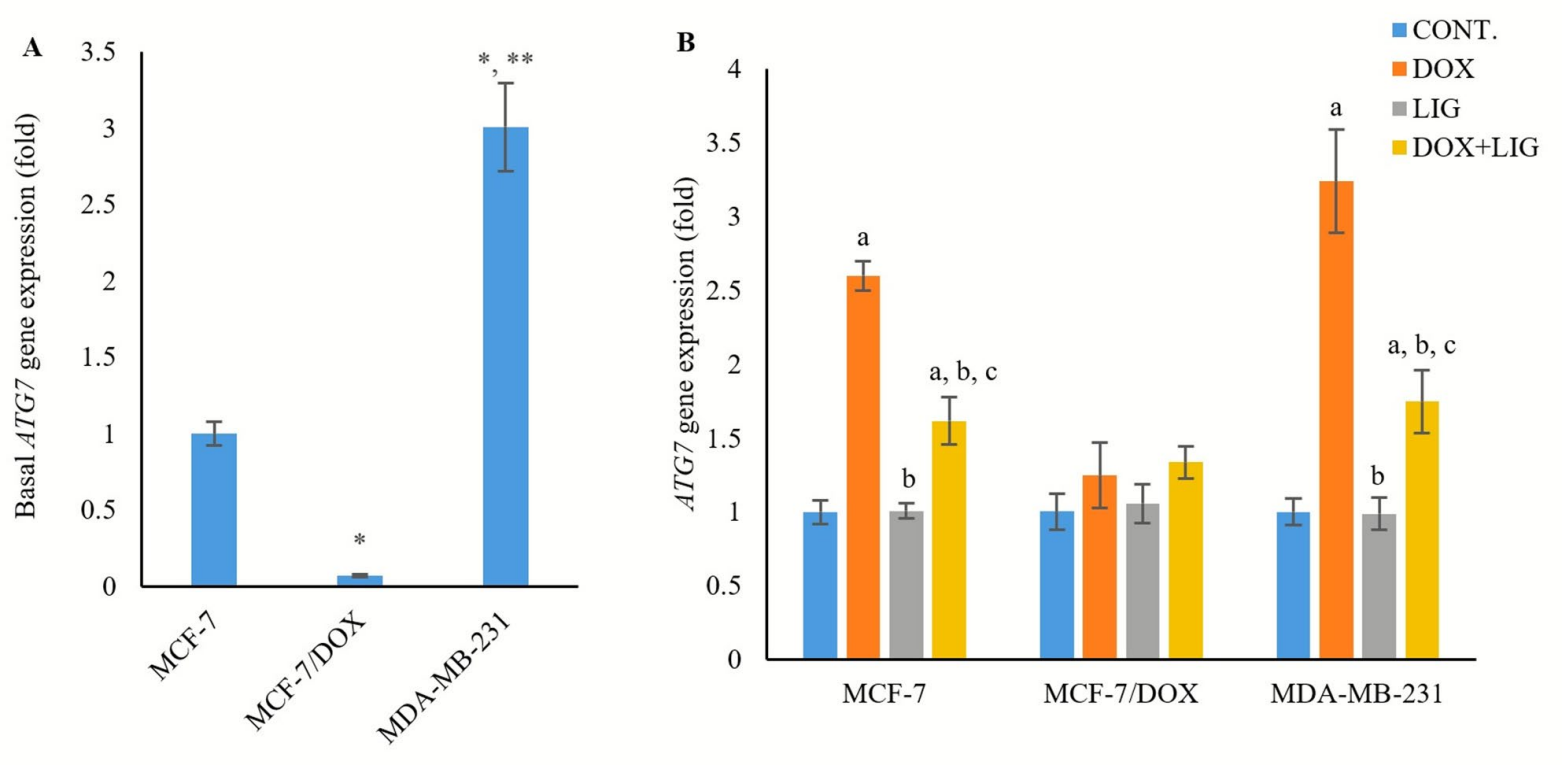
Basal expression of *ATG7* gene (**A**) and expression of *ATG7* gene in the treated cell line groups (**B**) evaluated by PCR after 72 h of treatment with (0.01 µM) Doxorubicin, (50 µM) Ligustilide, or a combination of both drugs. Values are presented as mean ± SD (*n* = 3 per group). CONT: Untreated control; DOX: Doxorubicin; LIG: Ligustilide. *: Statistically significant compared to MCF-7 untreated control; **: Statistically significant compared to MCF-7/DOX untreated control. a: Statistically significant compared to respective untreated control; b: Statistically significant compared to respective DOX-treated cells; c: Statistically significant compared to respective LIG-treated cells

Basal *ATG7* gene expression was significantly lower in MCF-7/DOX cells (0.07 ± 0.009 fold, *p* < 0.01) relative to the MCF-7 cells, but it was significantly higher in MDA-MB-231 cells (3.01 ± 0.29 fold, *p* < 0.001) relative to both MCF-7 and MCF-7/DOX cells (Fig. 5A).

In MCF-7 and MDA-MB-231 cells, DOX monotherapy significantly upregulated *ATG7* expression compared with the untreated controls (2.6 ± 0.1 fold and 3.24 ± 0.35 fold, respectively, *p* < 0.001). In contrast, LIG monotherapy had no significant effect on *ATG7* expression relative to the untreated controls (1.01 ± 0.05 fold and 0.99 ± 0.11 fold, respectively, *p* < 0.05). Moreover, (DOX + LIG) combined therapy significantly downregulated *ATG7* gene expression compared with DOX monotherapy in both MCF-7 and MDA-MB-231 cells (1.62 ± 0.16 fold and 1.75 ± 0.21 fold, respectively, *p* < 0.001), but significantly upregulated its expression compared with LIG monotherapy (*p* < 0.001 and *p* < 0.05, respectively). In MCF-7/DOX cells, neither DOX nor LIG, alone or in combination, significantly altered *ATG7* expression compared with the untreated control (1.25 ± 0.22 fold, 1.06 ± 0.13 fold and 1.34 ± 0.11 fold, respectively, *p* < 0.05) (Fig. 5B).

### Impact of DOX and LIG on estrogen receptor alpha

**Fig. 6:**
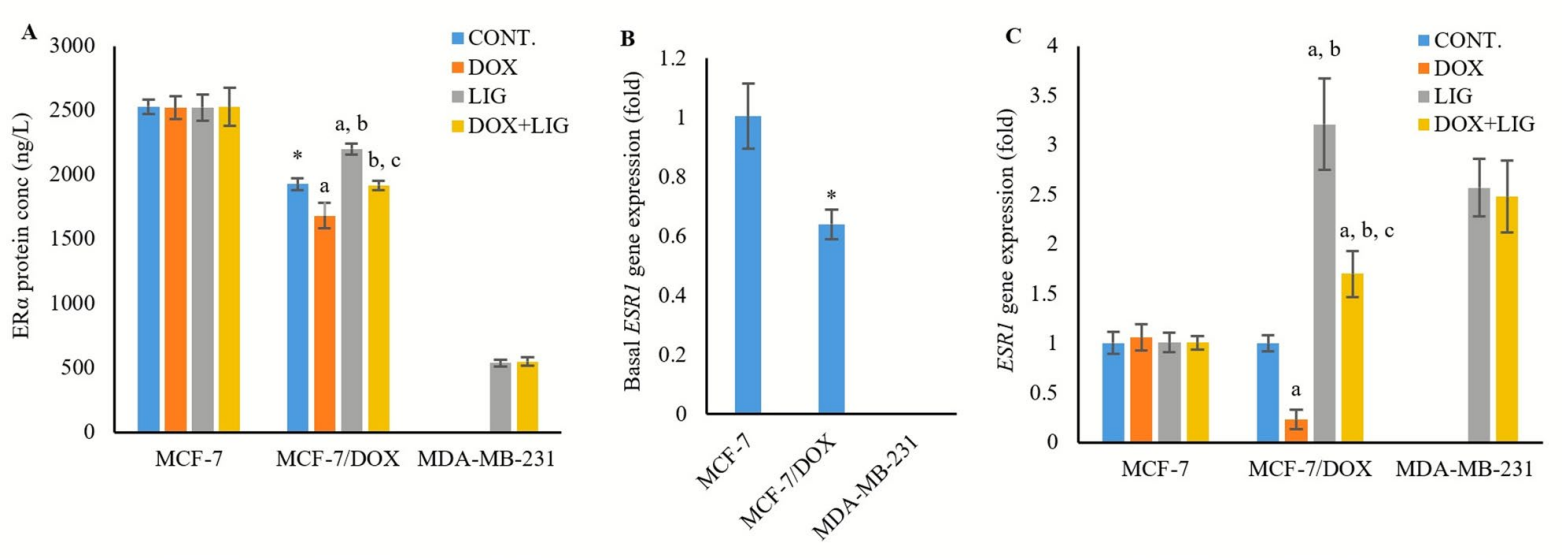
Concentration of ERα protein (**A**), basal expression of *ESR1* gene (**B**), and expression of *ESR1* gene in the treated cell line groups (**C**) evaluated by ELISA (for protein) and PCR (for gene) after 72 h of treatment with (0.01µM) Doxorubicin, (50µM) Ligustilide, or a combination of both drugs. Values are presented as mean ± SD (*n* = 3 per group). CONT: Untreated control; DOX: Doxorubicin; LIG: Ligustilide. *: Statistically significant compared to MCF-7 untreated control. a: Statistically significant compared to respective untreated control; b: Statistically significant compared to respective DOX-treated cells; c: Statistically significant compared to respective LIG-treated cells

Basal ERα protein and *ESR1* gene expression were significantly lower in MCF-7/DOX cells (1927.01 ± 45.64 ng/L, and 0.64 ± 0.05 fold, *p* < 0.001) compared with MCF-7 cells (2528.11 ± 54.744 ng/L). The ERα protein and mRNA were undetectable in MDA-MB-231 cells (Fig. 6A and B).

In MCF-7/DOX cells, DOX monotherapy reduced ERα protein and downregulated *ESR1* expression (1681.40 ± 98.45 ng/L, *p* < 0.01, and 0.23 ± 0.10 fold, *p* < 0.05), whereas LIG monotherapy significantly increased both protein and gene levels (2199.40 ± 42.16 ng/L, *p* < 0.01, and 3.21 ± 0.46 fold, *p* < 0.001), relative to the untreated controls. The combination treatment (DOX + LIG) significantly elevated ERα protein and *ESR1* expression compared with DOX monotherapy (1918.24 ± 36.64 ng/L, *p* < 0.01, and 1.70 ± 0.23 fold, *p* < 0.001), but significantly decreased both ERα protein and *ESR1* expression relative to LIG monotherapy (*p* < 0.01 and *p* < 0.001, respectively) (Fig. 6A and C).

In MDA-MB-231 cells, ERα protein and *ESR1* gene remained undetectable with DOX monotherapy, but were restored by LIG monotherapy (539.32 ± 27.07 ng/L and 2.57 ± 0.29 fold) and (DOX + LIG) combined therapy (550.31 ± 34.55 ng/L and 2.48 ± 0.36 fold), with no significant difference between these groups (*p* > 0.05). In contrast, MCF-7 cells showed no significant changes in ERα protein (2519.34 ± 88.77 ng/L, 2522.11 ± 101.46 ng/L and 2526.72 ± 147.79 ng/L) or *ESR1* gene expression (1.06 ± 0.13 fold, 1.01 ± 0.10 fold, and 1.007 ± 0.07 fold) after DOX, LIG, or combination treatment compared with untreated controls (*p* > 0.05) (Fig. 6A and C).

### Basal protein concentrations and gene expression levels of metastasis-associated protein 1 complex components in the three BC cell lines

**Fig. 7:**
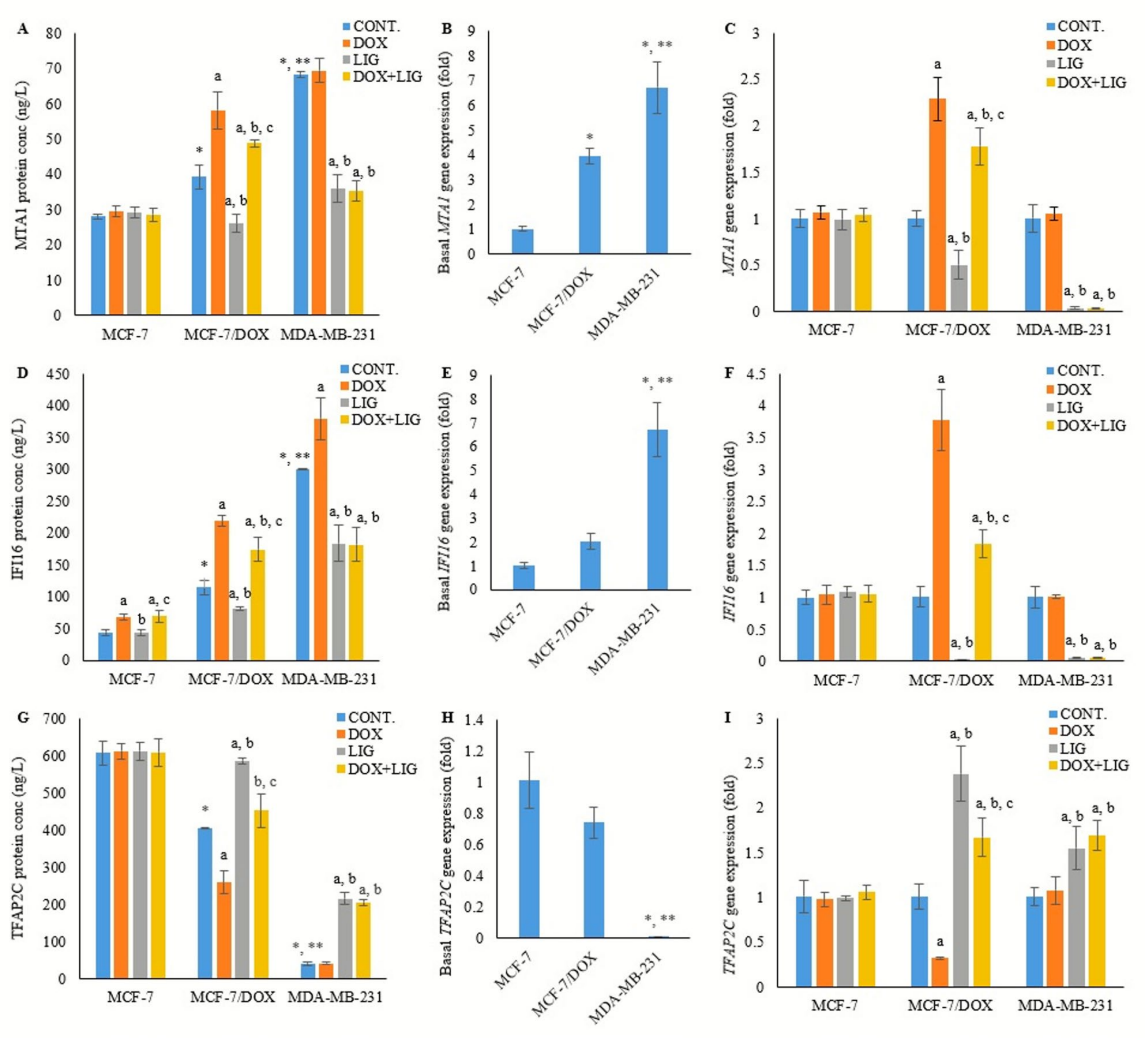
Concentration of MTA1 protein (**A**), basal expression of *MTA1* gene (**B**), expression of *MTA1* gene in the treated cell line groups (**C**), concentration of IFI16 protein (**D**), basal expression of *IFI16* gene (**E**), expression of *IFI16* gene in the treated cell line groups (**F**), concentration of TFAP2C protein (**G**), basal expression of *TFAP2C* gene (**H**), and expression of *TFAP2C* gene in the treated cell line groups (**I**), evaluated by ELISA (for protein) and PCR (for gene) after 72 h of treatment with (0.01 µM) Doxorubicin, (50 µM) Ligustilide, or a combination of both drugs. Values are presented as mean ± SD (*n* = 3 per group). CONT: Untreated control; DOX: Doxorubicin; LIG: Ligustilide. *: Statistically significant compared to MCF-7 untreated control; **: Statistically significant compared to MCF-7/DOX untreated control. a: Statistically significant compared to respective untreated control; b: Statistically significant compared to respective DOX-treated cells; c: Statistically significant compared to respective LIG-treated cells

Basal MTA1 protein and *MTA1* gene expression were significantly higher in MCF-7/DOX cells (39.27 ± 3.36 ng/L and 3.93 ± 0.32 fold, *p* < 0.01) compared with MCF-7 cells (27.97 ± 0.52 ng/L). In MDA-MB-231 cells, MTA1 protein and gene expression were further upregulated (68.27 ± 0.77 ng/L and 6.73 ± 1.04 fold) relative to both MCF-7 (*p* < 0.001) and MCF-7/DOX cells (*p* < 0.001 for protein, *p* < 0.01 for gene) (Fig. 7A and B).

Basal IFI16 protein was higher in MCF-7/DOX cells (114.57 ± 10.94 ng/L, *p* < 0.001), whereas *IFI16* gene expression showed no significant change (2.01 ± 0.33 fold, *p* > 0.05) compared with MCF-7 cells (43.56 ± 5.12 ng/L). In MDA-MB-231 cells, both basal IFI16 protein and gene expression were significantly higher (299.76 ± 1.16 ng/L and 6.70 ± 1.14 fold, *p* < 0.001) relative to MCF-7 and MCF-7/DOX cells (Fig. 7D and E).

Basal TFAP2C protein was lowered in MCF-7/DOX cells (404.55 ± 2.31 ng/L, *p* < 0.001), while basal *TFAP2C* expression remained significantly unchanged (0.74 ± 0.10, *p* > 0.05) compared with MCF-7 cells (607.11 ± 32.69 ng/L). In MDA-MB-231 cells, both basal TFAP2C protein and gene expression were significantly lower (40.33 ± 4.40 ng/L and 0.007 ± 0.0007 fold, *p* < 0.001) relative to MCF-7 and MCF-7/DOX cells (Fig. 7G and H).

### Differential expression of the metastasis-associated 1 complex components in MCF-7 cells subjected to DOX and LIG treatment

In MCF-7 cells, treatment with DOX, LIG, or their combination did not significantly alter MTA1 or TFAP2C at either the protein or gene expression levels compared with the untreated control (*p* > 0.05). MTA1 protein levels were measured at 29.63 ± 1.53 ng/L, 29.22 ± 1.50 ng/L, and 28.54 ± 1.89 ng/L, with corresponding *MTA1* expression values of 1.07 ± 0.070, 0.99 ± 0.11, and 1.04 ± 0.07 fold, following DOX, LIG, and combined treatment, respectively. Similarly, TFAP2C protein levels were measured at 611.44 ± 20.17 ng/L, 612.27 ± 24.17 ng/L, and 608.83 ± 36.90 ng/L, with corresponding *TFAP2C* expression values of 0.98 ± 0.08, 0.99 ± 0.03, and 1.06 ± 0.08 fold, following DOX, LIG, and combined treatment, respectively (Fig. 7A, C and G, and 7I).

In contrast, IFI16 protein levels were significantly increased following DOX monotherapy (67.20 ± 4.80 ng/L, *p* < 0.01), while no significant change was observed with LIG monotherapy (44.10 ± 5.05 ng/L, *p* > 0.05) relative to untreated control. In DOX + LIG combined therapy, IFI16 protein levels (69.53 ± 9.38 ng/L) did not differ significantly (*p* > 0.05) from DOX monotherapy but remained significantly higher than LIG monotherapy (*p* < 0.01). Despite these changes at the protein level, *IFI16* expression remained statistically unchanged across DOX, LIG, and combined treatment (1.05 ± 0.15 fold, 1.09 ± 0.09 fold, and 1.06 ± 0.14 fold, respectively) relative to the untreated control (*p* > 0.05) (Fig. 7D and F).

### Differential expression of the metastasis-associated 1 complex components in MCF-7/DOX cells subjected to DOX and LIG treatment

DOX monotherapy significantly increased MTA1 protein level and gene expression (58.13±5.27 ng/L and 2.29±0.23 fold, respectively, *p*<0.001), as well as IFI16 protein level and gene expression (218.44±8.62 ng/L and 3.77±0.48 fold, respectively, *p*<0.001) relative to untreated controls. Conversely, LIG monotherapy significantly reduced MTA1 protein level and gene expression (26.05±2.5 ng/L, *p*<0.01, and 0.50±0.15 fold, *p*<0.05, respectively) and reduced IFI16 protein level and gene expression (81±2.71 ng/L and 0.03±0.005 fold, respectively, *p*<0.05) compared to untreated controls. The combination of DOX and LIG significantly reduced MTA1 protein level and gene expression (48.72±1.05 ng/L and 1.78±0.20 fold, respectively, p<0.05), as well as IFI16 protein level and gene expression (174.26±18.79 ng/L, *p*<0.01 and 1.84±0.21 fold, *p*<0.001, respectively) relative to DOX monotherapy, while remaining higher than LIG monotherapy for both MTA1 and IFI16 protein and gene levels (*p*<0.001) (Figs. 7A, 7C, 7D, and 7F).

Conversely, DOX monotherapy significantly reduced TFAP2C protein and gene expression (258.27±31.0 ng/L, p<0.001 and 0.32±0.01 fold, p<0.05, respectively), whereas LIG monotherapy significantly increased TFAP2C protein level and gene expression (585.3±8.85 ng/L and 2.38±0.31 fold, respectively, p<0.001) relative to untreated control. Furthermore, (DOX+LIG) combined therapy significantly increased TFAP2C protein and gene expression (451.61±43.99 ng/L and 1.67±0.21 fold, respectively, p<0.001) relative to DOX monotherapy, but significantly decreased TFAP2C protein (p<0.001) and gene expression (p<0.05) relative to LIG monotherapy (Figs. 7G and 7I).

### Differential expression of the metastasis-associated 1 complex components in MDA-MB-231 cells subjected to DOX and LIG treatment

DOX monotherapy did not significantly alter MTA1 protein level or gene expression (69.42 ± 3.55 ng/L and 1.05 ± 0.07 fold, respectively, *p* > 0.05), nor TFAP2C protein level or gene expression (41.61 ± 1.95 ng/L and 1.07 ± 0.15 fold, respectively, *p* > 0.05) compared with the untreated control. In contrast, LIG monotherapy significantly reduced MTA1 protein level and gene expression (35.81 ± 3.93 ng/L and 0.04 ± 0.009 fold, respectively, *p* < 0.001), while significantly increasing TFAP2C at both protein and transcription levels (215.05 ± 16.67 ng/L, *p* < 0.001, and 1.55 ± 0.24 fold, *p* < 0.05, respectively) compared with the untreated control. The combination treatment (DOX + LIG) produced a profile comparable to LIG alone, with a significant reduction in MTA1 protein level and gene expression (35.27 ± 2.89 ng/L; 0.04 ± 0.001 fold; *p* < 0.001) and a significant increase in TFAP2C protein level and gene expression (205.38 ± 7.46 ng/L; 1.69 ± 0.17 fold; *p* < 0.05). These changes were significant relative to DOX monotherapy but not different from LIG monotherapy (*p* > 0.05) (Fig. 7A, C and G, and 7I).

For IFI16, DOX monotherapy significantly increased protein level (379.68 ± 33.24 ng/L, p < 0.05) without affecting gene expression (1.01 ± 0.03 fold, p > 0.05) compared with the untreated control. LIG monotherapy significantly reduced both IFI16 protein level (183.95 ± 29.0 ng/L, p < 0.01) and gene expression (0.063 ± 0.003 fold, p < 0.001) relative to untreated control. Similarly, the (DOX + LIG) combined therapy significantly reduced IFI16 protein and gene expression (181.55 ± 26.39 ng/L and 0.06 ± 0.009 fold, respectively, p < 0.001), showing significance versus DOX monotherapy but no significant difference compared with LIG monotherapy (p > 0.05) (Fig. 7D and F).

### Impact of DOX and LIG on long non-coding RNA H19 expression

**Fig. 8:**
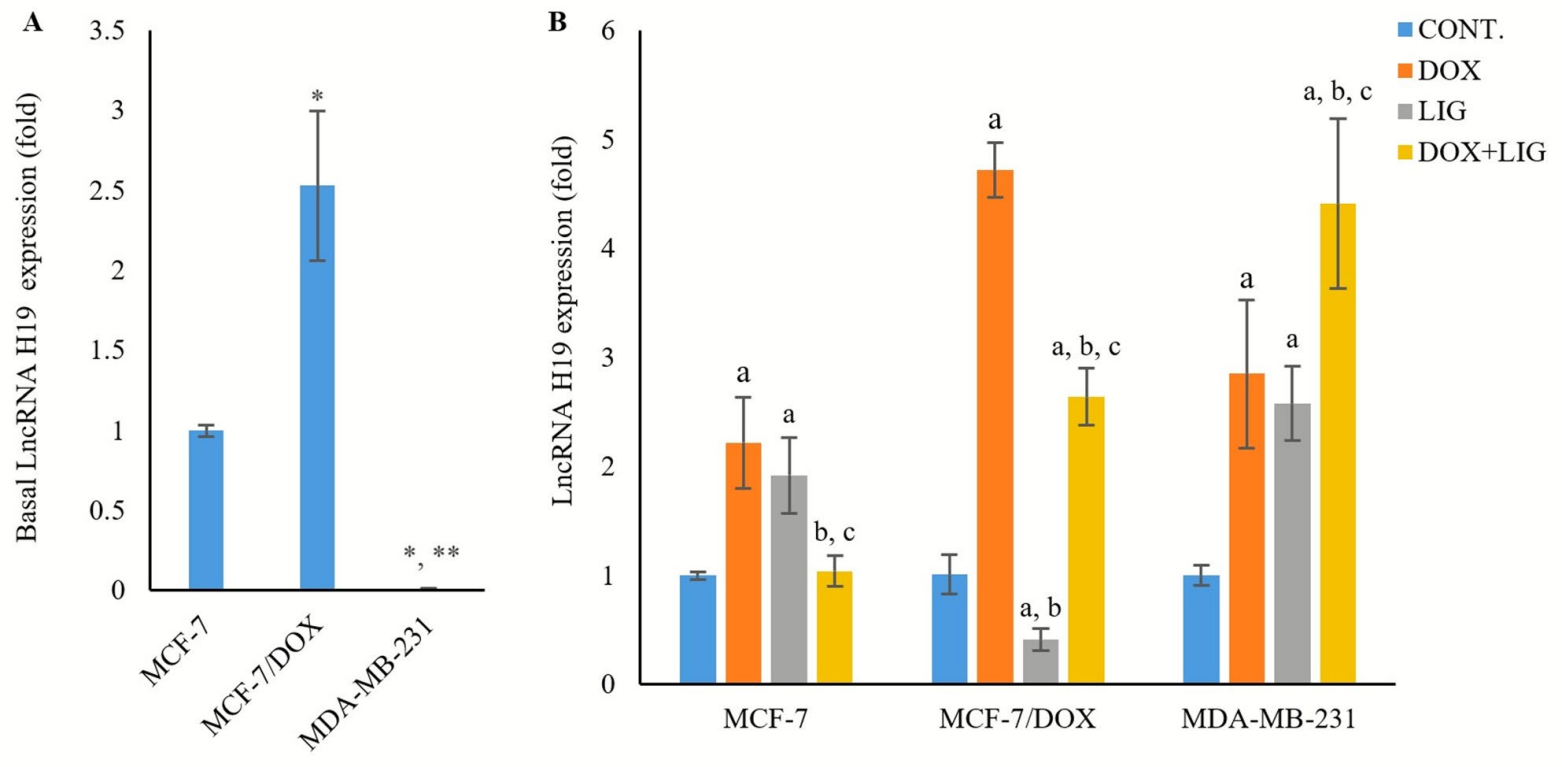
Basal expression of LncRNA H19 (**A**) and expression of LncRNA H19 in the treated cell line groups (**B**) evaluated by PCR after 72 h of treatment with (0.01µM) Doxorubicin, (50µM) Ligustilide, or a combination of both drugs. Values are presented as mean ± SD (*n* = 3 per group). CONT: Untreated control; DOX: Doxorubicin; LIG: Ligustilide. *: Statistically significant compared to MCF-7 untreated control; **: Statistically significant compared to MCF-7/DOX untreated control. a: Statistically significant compared to respective untreated control; b: Statistically significant compared to respective DOX-treated cells; c: Statistically significant compared to respective LIG-treated cells

Basal LncRNA H19 expression was significantly higher in MCF-7/DOX cells (2.53 ± 0.47 fold, *p* < 0.01) relative to MCF-7 cells, whereas it was significantly lower in MDA-MB-231 cells (0.014 ± 0.001 fold, *p* < 0.05 and *p* < 0.001) relative to MCF-7 and MCF-7/DOX cells, respectively (Fig. 8A).

DOX monotherapy significantly upregulated LncRNA H19 expression in all cell lines (2.22 ± 0.42 fold, *p* < 0.01 in MCF-7; 4.72 ± 0.25 fold, *p* < 0.001 in MCF-7/DOX; and 2.85 ± 0.68 fold, *p* < 0.05 in MDA-MB-231) relative to the untreated controls. LIG monotherapy also significantly upregulated LncRNA H19 expression in MCF-7 and MDA-MB-231 cells (1.92 ± 0.35 fold, and 2.58 ± 0.34 fold, respectively, *p* < 0.05), but significantly downregulated its expression in MCF-7/DOX cells (0.41 ± 0.10 fold, *p* < 0.05) relative to the untreated controls. Co-treatment with DOX and LIG produced cell line-dependent effects. In MCF-7 cells, LncRNA H19 expression was reduced to near-basal levels (1.04 ± 0.14 fold), significantly lower than with either DOX or LIG monotherapy (*p* < 0.01 and *p* < 0.05, respectively). In MCF-7/DOX cells, the combination attenuated DOX-induced upregulation (2.64 ± 0.26 fold vs. DOX, *p* < 0.001) but remained significantly higher than with LIG monotherapy (*p* < 0.001). Conversely, in MDA-MB-231 cells, co-treatment significantly increased LncRNA H19 expression (4.41 ± 0.78 fold, *p* < 0.05) compared with both monotherapies (Fig. 8B).

The highly significant results are based on cell models and should be interpreted as mechanistic evidence; further in vivo validation is needed. Protein expression was measured using ELISA, a reliable and quantitative method; these findings are preliminary, and Western blot validation will be performed in future studies to further strengthen the mechanistic evidence.

**Fig. 9:**
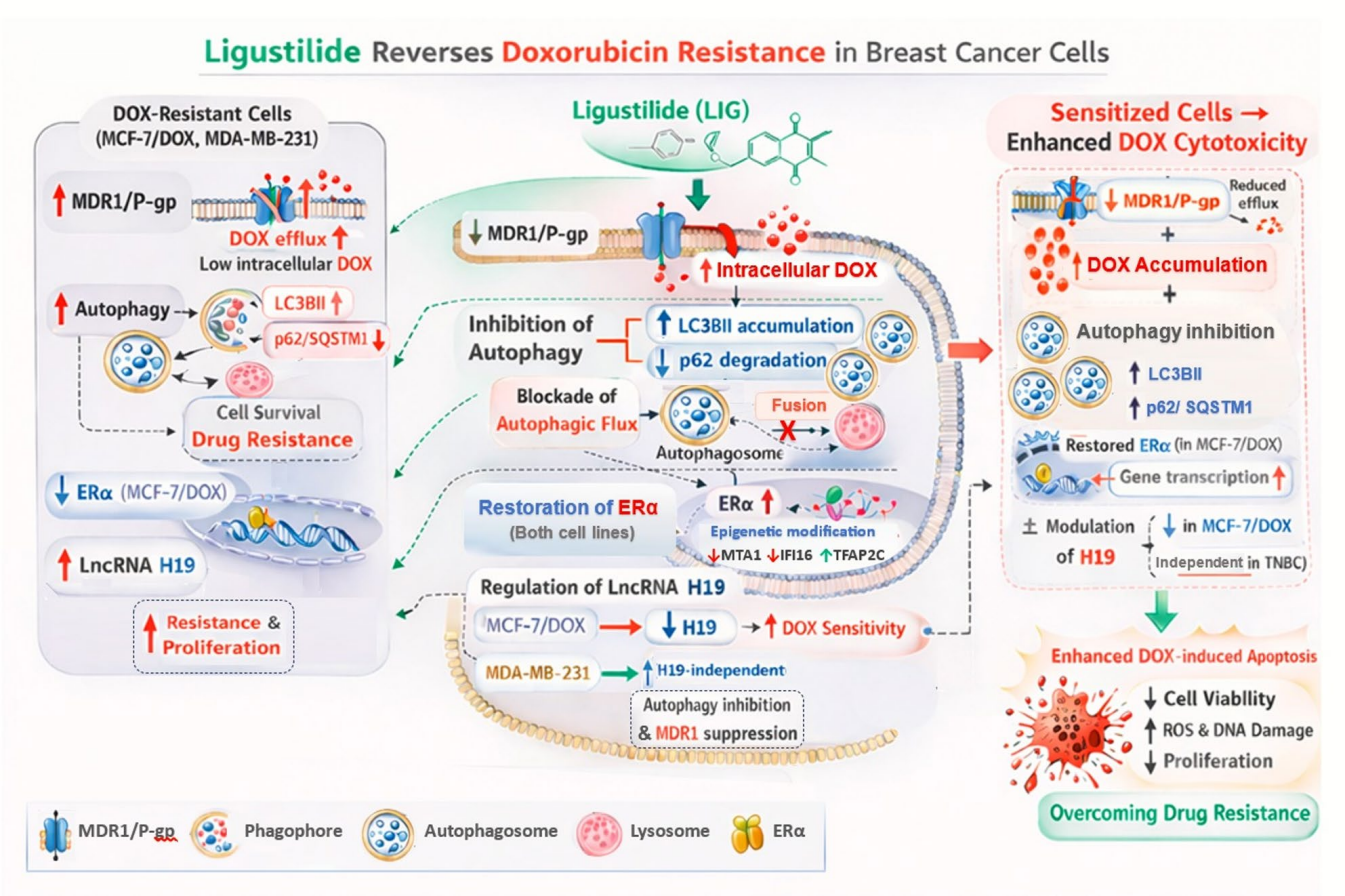
Schematic diagram of the proposed mechanism by which Ligustilide attenuates Doxorubicin resistance in breast cancer cells. The schematic illustrates the key findings in acquired (MCF-7/DOX) and intrinsic (MDA-MB-231) resistant BC models. Left panel (resistant phenotype): Resistant cells show elevated protective autophagy (↑LC3BII and ↓p62(, increased MDR1/P-gp–mediated drug efflux, reduced ERα expression in MCF-7/DOX, and elevated lncRNA H19 levels, all of which support survival and drug tolerance. Middle panel (direct effects of igustilide addition): Ligustilide (LIG) inhibits autophagy by interfering with autophagosome-lysosome fusion, reduces MDR1/P-gp expression, and restores ERα expression (in both cell lines) by epigenetic modification (↓MTA1, ↓IFI16, and ↑TFAP2C), and regulates LncRNA H19 in a subtype-dependent manner (↓ in MCF-7 and ↑ in MDA-MB-231). Right panel (relevant sensitization outcomes): Combined LIG + DOX therapy suppresses cytoprotective autophagy and reduces MDR1/P-gp level and activity. In MCF-7/DOX cells, resistance reversal appears linked to restoration of ERα and H19 modulation, whereas in MDA-MB-231 cells, sensitization primarily results from MDR1 inhibition and autophagy suppression. These effects enhance intracellular DOX retention, increase DNA damage and ROS generation, promote cytotoxicity, and restore chemosensitivity in resistant BC cells. The model provides an integrated conceptual framework based on the present findings rather than a definitive mechanistic hierarchy

The schematic diagram (Fig. 9) illustrates the proposed mechanism by which LIG enhances sensitivity, providing a clear overview of the molecular interactions identified in this study.

## Discussion

Autophagy contributes to BC progression and enhances resistance to anticancer therapies. Therefore, autophagy inhibition could be a prospective therapeutic target in overcoming BC resistance [6]. This research aimed to investigate the chemosensitizing potential of LIG in combination with DOX in three BC cell lines: sensitive (MCF-7), DOX-resistant (MCF-7/DOX), and TNBC (MDA-MB-231). In addition, the molecular pathways associated with chemoresistance and the potential involvement of LncRNA H19 were also evaluated.

Across all tested cell lines, both DOX and LIG exhibited dose-dependent cytotoxicity; however, different sensitivity patterns were observed. MDA-MB-231 cells exhibited intrinsic resistance to both drugs, whereas MCF-7/DOX cells were selectively resistant to DOX. Notably, co-treatment with LIG and DOX significantly enhanced cytotoxicity in both resistant models, whereas an antagonistic effect was observed in sensitive MCF-7 cells, highlighting the context-dependent nature of LIG’s effect. These findings indicate that LIG’s chemosensitizing effect is cellular dependent and should not be generalized across BC subtypes, emphasizing the importance of considering cell-type specificity when targeting autophagy to overcome drug resistance [17].

LIG increased both LC3BII and p62 levels across all three cell lines. LC3BII recruits to the autophagosome membrane, and p62 is involved in mediating the transport of ubiquitinated proteins in the autophagic process. The concurrent accumulation of both markers, together with the reversal of DOX-induced p62 depletion, is most consistent with impaired autophagic flux rather than enhanced autophagosome formation. This pattern suggests that LIG inhibits late-stage autophagy, potentially at the level of autophagosome-lysosome fusion, in line with previous studies [17, 23]. While these findings support impaired flux, they should be interpreted as mechanistic associations rather than definitive proof of causality, and further functional studies are needed to clarify the role of autophagy in LIG-mediated sensitization.

Our results also demonstrated that the effect of LIG is context-dependent, indicating the functional role of autophagy in each cell line. In MCF-7/DOX and MDA-MB-231 cells, where autophagy acts as a cytoprotective mechanism, LIG-mediated inhibition enhanced DOX-induced cytotoxicity. Conversely, in MCF-7 cells, autophagy inhibition by LIG reduced DOX efficacy, consistent with a cytotoxic role for DOX-induced autophagy. These observations align with previous studies demonstrating that blocking autophagy can attenuate chemotherapy-induced cytotoxic autophagy in MCF-7 cells [24, 25].

Our findings suggested a cytoprotective role of DOX-enhanced autophagy in MCF-7/DOX cells. Upon DOX entry to the tumor cells, mitochondria are affected prior to nuclear DNA binding, leading to oxidative damage through ROS generation [26]. This mitochondrial stress may trigger non-canonical autophagy, including mitophagy, which selectively removes DOX-damaged mitochondria and contributes to chemoresistance [26].

The *ATG7* gene plays a key role in autophagy by catalyzing two conjugation processes essential for autophagosome formation and expansion [27]. Autophagy can be either an *ATG7*-dependent (canonical) or *ATG7*-independent (non-canonical) process. Canonical autophagy is a nonselective process degrading bulk cytoplasmic components, while noncanonical autophagy can selectively target specific intracellular substrates [27].

Our work revealed that basal *ATG7* expression was highest in MDA-MB-231 cells relative to MCF-7 and MCF-7/DOX cells. Treatment with DOX upregulated *ATG7* in MCF-7 and MDA-MB-231 cells, while co-treatment with LIG downregulated *ATG7* expression in both cell lines, relative to DOX monotherapy. These findings suggest that cytotoxic autophagy in MCF-7 cells is mediated by *ATG7*-dependent canonical autophagy, consistent with a prior study showing that *ATG7* knockdown suppressed autophagy and promoted MCF-7 cell survival [28]. Similarly, in MDA-MB-231 cells, the intrinsic resistance and cytoprotective autophagy were *ATG7*-dependent, supporting the earlier studies demonstrating that *ATG7* silencing inhibited autophagy and attenuated the growth and invasiveness of MDA-MB-231 cells [29, 30].

In contrast, basal *ATG7* gene expression was lower in MCF-7/DOX cells relative to MCF-7. Previous studies have reported that higher basal autophagy in DOX-resistant MCF-7 cells can occur independently of *ATG7* expression or upstream regulators of canonical autophagy [26, 31]. Consistent with this, *ATG7* expression in our model remained unchanged in MCF-7/DOX cells following DOX and/or LIG treatment. Together with the observed modulation of autophagy-associated markers, these findings suggest that cytoprotective autophagy contributing to acquired resistance in MCF-7/DOX cells may proceed through *ATG7*-independent or non-canonical mechanisms. However, this interpretation should be considered a biologically supported hypothesis rather than definitive mechanistic proof, as functional manipulation of *ATG7* would be required for causal confirmation. Notably, this interpretation aligns with prior reports linking *ATG7* deficiency with the development of DOX resistance in MCF-7 cells [26, 28].

Chemoresistance was associated with the overexpression of the *MDR1* gene encoding the efflux transporter P-gp (ABCB1) [3]. In the present study, basal *MDR1* expression was detected in MDA-MB-231 and MCF-7/DOX cells, but not in MCF-7 cells, consistent with intrinsic resistance in TNBC cells and acquired resistance in MCF-7/DOX cells following prolonged DOX exposure as previously reported [3, 32]. DOX-induced resistance was evident, herein in both MCF-7/DOX and MDA-MB-231 cells, as reflected by upregulated *MDR1* expression following DOX monotherapy.

The addition of LIG to DOX enhanced cytotoxicity and significantly downregulated *MDR1* expression in both cell lines, compared with DOX monotherapy. This observation aligned with previous reports indicating that LIG inhibited P-gp efflux activity and downregulated *MDR1* expression at the mRNA level [33, 34]. Taken together, these findings supported the chemosensitizing effect of LIG, likely through suppression of *MDR1*-mediated drug efflux, thereby improving DOX efficacy in MCF-7/DOX and MDA-MB-231 cells.

Our findings also demonstrated that reduced ERα (protein and gene) expression in MCF-7/DOX was accompanied by higher basal autophagy with a cytoprotective profile compared with MCF-7 cells. This relationship suggested a functional shift in autophagy from a cytotoxic response in sensitive MCF-7 cells to a survival mechanism in the resistant phenotype. One plausible explanation was enhanced mitophagy during DOX resistance in MCF-7 cells, which might decrease mitochondrial ERα localization and activity [25, 35, 36]. Our results demonstrated that LIG monotherapy positively modulated ERα. Specifically, LIG monotherapy restored ERα (protein and gene) expression in MDA-MB-231 cells and further increased them in MCF-7/DOX cells, relative to untreated controls. Additionally, combining LIG with DOX enhanced ERα expression in both MCF-7/DOX and MDA-MB-231 cells compared with DOX monotherapy. These observations were further supported by the findings of *Ma et al.* [9]., , who first reported that LIG can restore ERα expression and transcriptional activity in TNBC cell lines.

Another explanation was provided by Chewchuk et al., who reported that acquiring DOX resistance in MCF-7 cells was associated with downregulation of ERα expression, leading to reduced levels of Bcl-2, an ERα-dependent protein that inhibits autophagy [35]. Interestingly, LIG appeared to play a dual inhibitory effect on autophagy in BC; first, by directly inhibiting autophagosome-lysosome fusion, similar to chloroquine (CQ); and second, by enhancing ERα expression, which subsequently upregulates Bcl-2, thereby reinforcing autophagy inhibition. This dual role may explain why LIG exhibits stronger autophagy suppression than CQ in resistant MCF-7 cells, as previously reported [17].

To explore the molecular mechanism behind LIG-induced and DOX-repressed ERα expression, was examined the levels of MTA1 along with its associated complex proteins, TFAP2C and IFI16. According to the current results, MDA-MB-231 cells exhibited the highest basal MTA1 and IFI16 levels and the lowest basal TFAP2C, followed by MCF-7/DOX cells and then MCF-7 cells. This finding is consistent with previous studies demonstrating that MTA1-mediated repression of ERα transcription is associated with resistance development in BC [9, 10, 12].

Co-treatment with LIG and DOX significantly reduced MTA1 and IFI16 levels while increasing TFAP2C levels in MCF-7/DOX and MDA-MB-231 cells compared with the DOX group. This effect likely reflects LIG’s ability to suppress autophagy and enhance or restore ERα expression in MCF-7/DOX and MDA-MB-231. LIG was considered as an epigenetic modulator that can reverse the epigenetic repression of ERα in TNBC mediated by the MTA1/IFI16/HDACs corepressor complex [9]. Consequently, autophagy inhibition by LIG was accompanied by favorable effects on drug resistance and ERα expression in MCF-7/DOX and MDA-MB-231 cells.

In contrast, MCF-7 cells showed no alteration in the level of ERα and MTA1 complex components following LIG and/or DOX treatment, except for IFI16 protein. Interestingly, although IFI16’s mRNA levels remained unchanged, its protein levels increased after DOX mono- or combined therapy, consistent with previous reports indicating that IFI16 regulates DNA damage response in BC cells; its protein levels increase after chemotherapy despite stable mRNA levels, suggesting regulation *via* post-translational modifications [37, 38].

To clarify, although LIG reduced DOX-induced cytotoxicity in MCF-7 cells, IFI16 protein remained elevated, suggesting a stress response rather than a direct indicator of cell death. IFI16 primarily mediates DNA-damage sensing and innate immune signaling [37, 38]. DOX continues to induce DNA lesions even when cytotoxicity is reduced, and inhibition of cytotoxic autophagy may limit the clearance of damaged nuclear components and protein aggregates, thereby prolonging DNA-damage signaling. Because IFI16 is regulated through post-translational mechanisms [37], autophagy inhibition may reduce its turnover and contribute to sustained protein accumulation. These findings indicate that IFI16 elevation reflects persistent genomic stress rather than enhanced resistance.

LncRNA H19 has been proposed as a diagnostic and prognostic biomarker in BC and a potential therapeutic target [39]. Based on our results, MCF-7 cells exhibited higher basal LncRNA H19 expression than MDA-MB-231 cells, consistent with previous studies describing LncRNA H19 as an estrogen-regulated gene that supports estrogen-dependent proliferation and survival [39–41]. These findings correspond to the luminal trait of MCF-7 cells and the hormone-independent nature of MDA-MB-231 cells.

DOX monotherapy upregulated LncRNA H19 expression in MCF-7 and MDA-MB-231 cells, suggesting activation of DNA damage-response pathways and consistent with the role of LncRNA H19 in repairing DOX-induced DNA double-strand breaks, as reported in previous studies [41]. Co-treatment with DOX and LIG modulated LncRNA H19 expression in a cell line-dependent manner, reflecting the associated cytotoxic effects. In MCF-7 cells, LIG reduced DOX cytotoxicity and was associated with downregulation of LncRNA H19, suggesting interference with DOX-induced cytotoxic autophagy. In contrast, in MDA-MB-231 cells, the combination enhanced cytotoxicity and was accompanied by upregulation of LncRNA H19, consistent with inhibition of cytoprotective autophagy.

We also observed that basal LncRNA H19 was higher in MCF-7/DOX cells than in sensitive MCF-7 cells, supporting its role in mediating acquired DOX resistance in MCF-7 cells, as reported in previous studies, through regulation of DNA repair factors, including poly (ADP-ribose) polymerase and CUL4A [42, 43]. DOX monotherapy in MCF-7/DOX cells further upregulated LncRNA H19 expression, accompanied by enhanced autophagy, elevated *MDR1* expression, and reduced ERα expression, consistent with previous findings that LncRNA H19 mediates endocrinal resistance *via* enhancing autophagy in ER + ve BC [40, 44]. Moreover, LncRNA H19 has been shown to prevent ERα downregulation induced by endocrine therapy and to regulate ERα expression at both transcript and protein levels in BC cell lines resistant to endocrine therapy [14, 45].

Importantly, LIG co-treatment with DOX in MCF-7/DOX cells downregulated LncRNA H19 expression, accompanied by downregulated *MDR1*, upregulated ERα, and suppressed autophagy. These results demonstrated that LncRNA H19 contributes to acquired DOX resistance in ER + ve BC, but does not appear to mediate intrinsic resistance in TNBC. Although modulation of LncRNA H19 was observed, our data suggest that its contribution to LIG-mediated chemosensitization is likely cell-type dependent. Particularly, in the TNBC model, LIG-mediated enhancement of cytotoxicity appears to occur independently of lncRNA H19 regulation, indicating that additional mechanisms, such as impaired autophagic flux and stress-response signaling, may predominate in this context.

Our findings are based on MCF-7/DOX (ER + ve acquired resistance) and MDA-MB-231 (intrinsic TNBC resistance) cells, enabling us to evaluate how LIG modulates resistance-related pathways in these distinct BC contexts. This approach also allowed us to determine the observed molecular effects are subtype-dependent rather than universally conserved. Accordingly, the effects of LIG should not be generalized to all TNBCs, and additional studies using other resistant TNBC models are warranted. In addition, although LIG modulated autophagy, ERα, *MDR1*, and LncRNA H19, these relationships remain associative rather than causal, and targeted functional studies will be required to establish mechanistic hierarchy.

## Conclusion

Autophagy suppression by LIG could be a potential strategy for combating BC resistance. In both DOX-resistant and triple-negative breast cancer cell lines, LIG when combined with DOX as adjuvant therapy exhibited improved chemosensitivity, which was mediated by slowed-down autophagy, induced ERα expression, and decreased multidrug resistance. LncRNA H19 may contribute to acquired DOX-resistance in ER + ve BC and could be a target of therapy by anticancer drugs. However, the upregulation of LncRNA H19 in triple-negative BC cells upon co-treatment with LIG and DOX requires further explanation. More research is required to evaluate LIG’s therapeutic effectiveness in modulating epigenetic mechanisms and inhibiting autophagy to sensitize resistant cancers to cytotoxic drugs.

## Data Availability

The datasets generated and analyzed during the current study are available from the corresponding author on reasonable request.

## Abbreviations

ANOVA: Analysis of variance
ATG7: Autophagy-related 7
BC: Breast cancer
cDNA: Complementary DNA
CQ: Chloroquine
Ct: Threshold cycle
DMEM: Dulbecco’s Modified Eagle Medium
DMSO: Dimethyl sulfoxide
DOX: Doxorubicin
ELISA: Enzyme-linked immunosorbent assay
ER: Estrogen receptor
ERα: Estrogen receptor alpha
ER+ve: Estrogen receptor-positive
ER-ve: Estrogen receptor-negative
ESR1: Estrogen receptor alpha gene
FBS: Fetal bovine serum
GAPDH: Glyceraldehyde-3-phosphate dehydrogenase
HDACs: Histone deacetylases
HER2: Human epidermal growth factor receptor 2
IC50: Half maximal inhibitory concentration
IFI16: IFN-γ-inducible protein 16
LIG: Ligustilide
LncRNA: Long noncoding RNA
LC3BII: Microtubule-associated protein 1 light chain 3 beta II
MDR1: Multidrug resistance gene 1
MTA1: Metastasis-associated protein 1
NuRD: Nucleosome remodeling and deacetylase
P-gp: P-glycoprotein
PR: Progesterone receptor
qRT-PCR: Quantitative reverse-transcription polymerase chain reaction
ROS: Reactive oxygen species
SD: Standard deviation
SPSS: Statistical Package for the Social Sciences
SRB: Sulforhodamine B
TCA: Trichloroacetic acid
TFAP2C: Transcription factor AP-2 g
TNBC: Triple-negative breast cancer
